# Quantitative insights into stroke recovery utilizing delayed vessel ratio from color-coded multiphase computed tomography angiography

**DOI:** 10.3389/fneur.2025.1568717

**Published:** 2025-06-18

**Authors:** Yu Lin, Xiaoxiao Zhang, Zhen Xing, Xiefeng Yang, Qingwen Tong, Shaomao Lv, Jinan Wang, Dairong Cao

**Affiliations:** ^1^Department of Radiology, The First Affiliated Hospital of Fujian Medical University, Fuzhou, China; ^2^Department of Radiology, Zhongshan Hospital Affiliated to Xiamen University, School of Clinical Medicine of Fujian Medical University, Xiamen, China; ^3^Xiamen Radiology Quality Control Center, Zhongshan Hospital Affiliated to Xiamen University, School of Clinical Medicine of Fujian Medical University, Xiamen, China; ^4^Department of Health Examination, Xiamen Humanity Hospital Fujian Medical University, Xiamen, China; ^5^Department of Radiology, National Regional Medical Center, Binhai Campus of the First Affiliated Hospital of Fujian Medical University, Fuzhou, China; ^6^Fujian Provincial Key Laboratory of Precision Medicine for Cancer, The First Affiliated Hospital of Fujian Medical University, Fuzhou, China; ^7^Key Laboratory of Radiation Biology of Fujian Higher Education Institutions, The First Affiliated Hospital of Fujian Medical University, Fuzhou, China

**Keywords:** computed tomography angiography, collateral circulation, stroke, delayed vessel ratio, venous outflow

## Abstract

**Background and objective:**

The color-coded multiphase computed tomography angiography (cmCTA) is an accredited technique that employs color-coding to visually depict the temporal dynamics of collateral blood flow in patients with acute ischemic stroke (AIS). This research aimed to assess the quantification of cmCTA in AIS patients for characterizing arterial and venous collateral flow, and predicting functional outcomes.

**Methods:**

A retrospective study was performed on a consecutive cohort of AIS patients with large vessel occlusion who underwent cmCTA scan and reconstruction. Collateral ratio and delayed vessel ratio (DVR) were determined through semi-automatic delineation and calculation on the anterior cerebral artery regions and Alberta Stroke Program Early CT (ASPECT) Score regions of cmCTA maps. Deep venous outflow (DVO) and superficial venous outflow (SVO) scores were assessed using a 6-point scale. Logistic regression and propensity score were applied to confounding factors adjustment and model construction. Receiver operating characteristic curve, calibration curve, and decision curve analysis were utilized to evaluate the prediction model of functional independence and excellent recovery.

**Results:**

Well-developed arterial collaterals as depicted by low DVR and adequate venous collaterals as indicated by high DVO or SVO were correlated with better outcomes (All *p* < 0.001). Adjusted DVR showed areas under the curve of 0.81–0.90 for predicting functional independence and excellent recovery. Adjusted DVO showed areas under the curve of 0.88 for predicting functional independence and excellent recovery. Each prediction model demonstrated good precision and net benefit.

**Conclusion:**

The application of DVR and other parameters in cmCTA offers a quantitative perspective on the conventional ASPECT scoring scheme utilizing grayscale CT images. DVR from cmCTA may enhance pre-treatment collateral assessment and post-treatment outcome prediction in AIS, facilitating informed treatment decisions.

## 1 Introduction

Acute ischemic stroke (AIS) caused by proximal occlusion in the intracranial cerebral arteries is a significant contributor to global morbidity and mortality, frequently associated with unfavorable clinical prognoses (1–3). Endovascular treatment (EVT) has emerged as a promising therapeutic approach for AIS patients with large vessel occlusion (LVO), particularly when administered within the appropriate therapeutic time-frame (4–7). The efficacy of EVT is significantly influenced by the pre-treatment evaluation using computed tomography angiography (CTA), which offers essential insights into the arterial and venous collateral status of ischemic brain tissue (8–13). Mayer et al. introduced a protocol mandating collateral assessment within 24 h of symptom onset for all patients with LVO-AIS, irrespective of initial stroke severity, in order to assess clinical outcomes following intervention (8). Furthermore, Madelung’s findings suggest that the presence of leptomeningeal collaterals can serve as a prognostic indicator for both functional outcomes and mortality in individuals who have experienced AIS with middle cerebral artery occlusion, irrespective of whether they have undergone EVT (12).

Conventionally, single-phase CTA (sCTA) has been widely employed in the routine clinical assessment of arterial collateral status and venous drainage patterns in AIS patients (4, 9, 14). Nevertheless, sCTA has limitations in accurately evaluating collateral blood flow due to its reliance on a single acquisition phase, which may not adequately capture the dynamic nature of collateral circulation (4, 14). Furthermore, the timing of sCTA acquisition can impact the assessment of collateral status, potentially introducing biases in clinical outcome prediction, even in large-scale clinical investigations (4, 9).

In response to these constraints, multiphase CTA (mCTA) has been developed, involving sequential acquisitions with short time intervals to enable a thorough assessment of collateral circulation at both arterial and venous levels over a period of time (6, 7, 15, 16). Studies have demonstrated that mCTA enhances the precision of arterial level collateral grading or venous outflow profiling compared to sCTA and can better predict clinical outcomes in AIS patients (10, 11, 15, 16). Nonetheless, the interpretation of mCTA images still relies predominantly on visual analysis, which can be subjective and prone to errors.

The quantitative collateral ratio (CR) can be calculated for both sCTA and selected phases of mCTA by determining the ratio of the enhanced vessel volume on the occluded side to the contrast-enhanced vessel volume on the healthy side (17–19). This method of assessing CR is considered to be robust and reproducible, providing quantitative collateral blood flow information that is comparable to that obtained from CT perfusion imaging (17, 20). Nevertheless, it is important to note that CR is a phase-specific parameter, and its diagnostic efficacy may be influenced by the scanning protocol and the phase selected for analysis.

The color-coded mCTA (cmCTA) has recently been developed as an accredited technique that employs color-coding to visually depict the temporal dynamics of collateral perfusion on mCTA images (21). This method shows promise in improving the precision and consistency of collateral grading, while also offering a more intuitive representation of arterial or venous collateral status for healthcare professionals (21–23). In this protocol, cerebral vessels exhibiting pronounced enhancement during the peak artery phase are highlighted in red, whereas vessels with delayed enhancement are depicted in green or blue, thereby enabling the quantification of the proportion of delayed vessels or delayed vessel ratio (DVR). It is hypothesized that such quantitative parameters, in conjunction with semi-quantitative venous outflow scores, will offer more precise and objective insights into cerebral collateral status. However, previous studies on cmCTA have focused on subjective evaluation through visual assessment of color distribution of cerebral vessels, lacking a quantitative evaluation framework.

Our study seeks to explore two quantitative parameters obtained from cmCTA maps (CR and DVR) and their correlation to clinical outcomes in AIS patients. Our specific objective is to evaluate the predictive capabilities of arterial-level collateral parameters and venous-level collateral scores in determining functional independence and excellent recovery at the 3-month follow-up.

## 2 Materials and methods

### 2.1 Study design and participants

We collected the clinical and imaging data of patients with consecutive clinically suspected AIS with anterior circulation LVO in our University Hospital from November 2018 to January 2022. The study was approved by the Institutional Review Board of both the First Affiliated Hospital of Fujian Medical University and Zhongshan Hospital Affiliated to Xiamen University. All methods were performed in accordance with the relevant guidelines and regulations as set by the approving institutions in a standardized manner. Written informed consent was given by all participants or their relatives.

The inclusion criteria were as follows: (1) Clinical diagnosis of AIS with the presence of anterior circulation LVO. (2) Patients age ≥ 18 years old. (3) Onset time ≤ 72 h; (4) All patients underwent pre-treatment multimodal CT [non-contrast computed tomography (NCCT) and mCTA]; (5) Complete baseline clinical data [such as demographic information, baseline National Institute of Health Stroke Scale (NIHSS) score, clinical history and comorbidities]; and (6) Complete clinical follow-up data [such as modified Rankin Scale (mRS) score on day 90].

The exclusion criteria were as follows: (1) Intracranial hemorrhage. (2) Posterior circulation LVO AIS. (3) Allergic to iodine contrast media. (4) Serious complications. (5) Poor image quality or incomplete image data.

### 2.2 CT examination

NCCT scanning parameters were as follows: tube voltage = 140 kV, tube current adopts automatic modulation technology, layer thickness = 5 mm/0.625 mm, Adaptive Statistical Iterative Reconstruction Veo (ASIR-V) = 40%, noise index (NI) = 5.

The mCTA examination included three-phase image scanning of arterial peak phase, venous peak phase and late venous phase. 40 ~ 50 mL of iodine contrast agent (Iomeron 400, Bracco, Italy) was injected at a rate of 4.5 ~ 5 mL/s before the scan, then equivalent saline was injected at the same rate. The scanning parameters of each phase were as follows:

Peak arterial phase: The intelligent trigger technology (threshold: 120 Hu) is used for scanning. Tube voltage = 100 kV, the tube current adopts the automatic modulation technology, layer thickness = 0.625 mm, ASIR-V = 40%, NI = 5.

Peak venous phase: The scan was triggered after a delay of 10 s after the peak arterial phase scan. Tube voltage = 100 kV, tube current = 300 mA, slice thickness = 1.25 mm, ASIR-V = 40%, NI = 5.

Late venous phase: The scan is triggered after a delay of 8 s after the peak venous phase scan. Tube voltage = 100 kV, tube current = 300 mA, layer thickness = 1.25 mm, ASIR-V = 40%, NI = 5.

### 2.3 Color-coded mCTA reconstruction

All NCCT and mCTA images were post processed on the GE Revolution CT workstation (AW 4.7, GE Healthcare, United States) through the image analysis software package (FastStroke, GE Healthcare, United States). The MIP images of cross-sectional ColorViz map (layer thickness = 35 mm, color saturation = 100%) were generated. The images were adjusted appropriately to ensure left–right symmetry.

### 2.4 Quantitative arterial level collateral parameters

**Figure 1:**
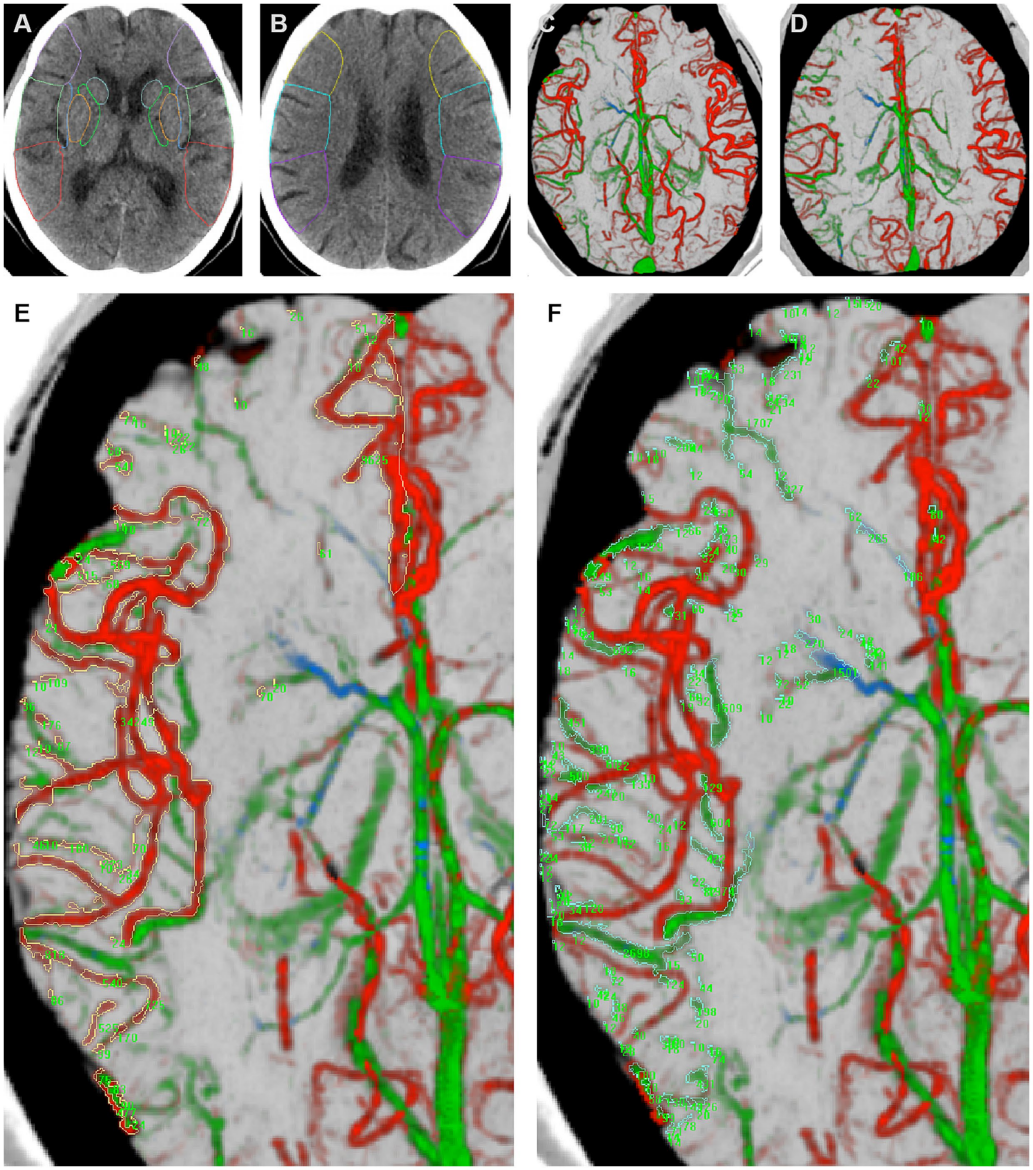
Based on the Alberta Stroke Program Early CT Score regions **(A,B)** and anterior cerebral artery regions of ColorViz map **(C,D)**, the anterior-circulation “red” vessels (significantly enhanced in the peak artery phase) and “delayed” vessels (“green” and “blue” vessels, significantly enhanced in the peak venous phase and late venous phase) on the affected side of patients with acute ischemic stroke were delineated **(E,F)**.

We subsequently utilized the post-processing software Image Pro Plus (version 7.0, Media Cybernetics, United States) to analyze the cmCTA maps, focusing on the anterior cerebral artery (ACA) regions and the automatic segmented Alberta Stroke Program Early CT Score (ASPECT) regions. By examining variations in color levels, vessels were categorized as “red” (indicating significant enhancement during the peak arterial phase) and “delayed” (comprising “green” and “blue” vessels, which showed significant enhancement during the peak venous and late venous phases). These vessels were semi-automatically delineated by two radiologists within ACA and ASPECT regions (Figure 1). The areas of “red” vessels, “delayed” vessels, and the total vessel area on both the affected and contralateral sides were quantified in pixels using the “count/size” module, with results averaged across the two measurements.

The arterial level collateral parameters including CR, DVR and relative delayed vessel (rDV) of the affected side were calculated, respectively. Meanwhile, the contralateral DVR (cDVR) was calculated. The specific calculation formulas were as follows:

(1) CR = total vessel area on the affected side/total vessel area on the unaffected side × 100%. (2) DVR = delayed vessel area on the affected side/total vessel area on the affected side × 100%. (3) cDVR = delayed vessel area on the contralateral unaffected side/total vessel area on the contralateral unaffected side × 100%. (4) rDV = delayed vessel area on the affected side/delayed vessel area on the contralateral unaffected side × 100%.

### 2.5 Semi-quantitative venous level collateral score

Using a 6-point scale ranging from 0 (worst) to 6 (best), two radiologists independently assessed collateral circulation at the venous level on the ColorViz map without knowing the patient’s prognosis. In cases of inconsistent results, discrepancies were resolved through negotiation. Specifically, the degree of venous filling (ranging from 0 to 3 points) and the degree of phase delay (ranging from 0 to 3 points) were evaluated separately, and the combined total score was calculated. Scores for deep venous outflow (DVO) and superficial venous outflow (SVO) were assessed, respectively (Supplementary material).

### 2.6 Clinical outcome

The primary endpoint of this study was the functional independence of AIS patients on the 90th day of clinical follow-up based on a blinded comprehensive assessment. Functional independence was defined as a mRS score ≤ 2. Secondary clinical endpoints included excellent recovery at the 90th day of follow-up. Excellent recovery was defined as an mRS score of 0 or 1, indicating nearly complete restoration of neurological function. Symptomatic intracranial hemorrhage (SICH) events during hospitalization were also recorded.

### 2.7 Statistical analysis

Intraclass Correlation Coefficient (ICC) was used to evaluate the interobserver agreement of our study. ICC values greater than 0.75 indicated excellent consistency. For all quantitative data, *t*-test or Mann–Whitney *U*-test was used for comparison between groups. For all qualitative data, Chi-square test or Fisher exact test was used for comparison between groups. The logistic regression was used to identify the risk factors and calculate the odds ratio. The propensity score was applied to correct confounding factors and optimize the prediction model. Receiver operating characteristic (ROC) curve, calibration curve and decision curve analysis (DCA) were used to evaluate the prediction efficiency of each prediction model. The De-Long test was used to compare the area under the curve (AUC) of ROC curve of each model. A *p* value less than 0.05 will be defined as a significant difference.

## 3 Results

### 3.1 Demographic and clinical characteristics

A total of 169 AIS patients were finally included in the study, with 76 (44.97%) patients classified as functionally dependent and 93 (55.03%) patients as functionally independent (Table 1). Among them, a total of 58 (34.32%) patients achieved excellent recovery after treatment. Age was comparable between the two groups, with a median of 64 years (IQR: 56, 72) in the overall population. The male gender was prevalent in both the functionally dependent group (65.79%) and the functionally independent group (78.49%).

**Table 1:** The demographic and clinical characteristics of AIS patients.

Characteristics	Total (*n* = 169)	Functional dependence (*n* = 76)	Functional independence# (*n* = 93)	*p*-value
Age (years), *median (IQR)*	64.00 (56.00, 72.00)	66.00 (53.00, 72.00)	64.00 (58.00, 72.00)	0.974
Male gender, *n (%)*	123 (72.78)	50 (65.79)	73 (78.49)	0.065
Baseline NIHSS, *median (IQR)*	4.00 (2.00, 11.00)	10.00 (3.75, 14.00)	3.00 (1.00, 5.00)	<0.001*
Comorbidities, *n (%)*				
Hypertension	117 (69.23)	49 (64.47)	68 (73.12)	0.226
Diabetes	54 (31.95)	19 (25.00)	35 (37.63)	0.080
Hyperlipidemia	58 (34.32)	25 (32.89)	33 (35.48)	0.724
Atrial fibrillation	45 (26.63)	25 (32.89)	20 (21.51)	0.096
History of smoking	72 (42.60)	34 (44.74)	38 (40.86)	0.612
History of alcohol abuse	44 (26.04)	19 (25.00)	25 (26.88)	0.782
Culprit vessel, *n (%)*				
ICA	41 (24.26)	18 (23.68)	23 (24.73)	0.874
ACA	4 (2.37)	2 (2.63)	2 (2.15)	1.000
MCA	109 (64.50)	46 (60.53)	63 (67.74)	0.329
ICA + MCA	15 (8.88)	10 (13.16)	5 (5.38)	0.077
Reperfusion therapy, *n (%)*				
EVT	41 (24.26)	26 (34.21)	15 (16.13)	0.006*
Thrombolysis	17 (10.06)	6 (7.89)	11 (11.83)	0.398
Thrombolysis + EVT	12 (7.10)	7 (9.21)	5 (5.38)	0.334
SICH	24 (14.20)	17 (22.37)	7 (7.53)	0.006*

AIS, acute ischemic stroke; IQR, interquartile range; NIHSS, National Institute of Health Stroke Scale; ICA, internal carotid artery; ACA, anterior cerebral artery; MCA, middle cerebral artery; EVT, endovascular thrombectomy; SICH, symptomatic intracranial hemorrhage. #Functional independence is defined as modified Rankin scale score = 0 ~ 2. *Significant differences.

The baseline NIHSS scores were significantly higher in the functionally dependent group compared to the functionally independent group (*p* < 0.001). Hypertension was the most prevalent condition, affecting 117 (70.31%) AIS patients. However, no significant difference in the prevalence of hypertension or other comorbidities was observed between the two groups (*p* > 0.05). The distribution of culprit vessels and the proportions of patients who received thrombolysis did not differ significantly between the two groups. However, within the functional dependent group, a higher proportion of AIS patients received EVT treatment and experienced SICH events.

### 3.2 Collateral parameters analysis

The ICCs of all collateral quantitative and semi-quantitative parameters were greater than 0.75, indicating excellent consistency.

The assessment of arterial level collateral parameters revealed several significant differences between the functionally dependent and independent groups (Table 2). Specifically, the DVR and rDV values were significantly higher in the functionally dependent group compared to the functionally independent group (both *p* < 0.001). Low DVR was a predictive factor for functional dependence and excellent recovery before and after propensity scores adjustment (Tables 3, 4). Additionally, rDV was a predictive factor for functional dependence before and after propensity scores adjustment.

**Table 2:** The arterial level and venous level collateral parameters derived from color-coded mCTA maps of AIS patients.

Collateral parameters	Total (*n* = 169)	Functional dependence (*n* = 76)	Functional independence^#^ (*n* = 93)	*p*-value
Arterial level collateral				
CR, *median (IQR)*	0.83 (0.66, 1.01)	0.83 (0.62, 0.99)	0.84 (0.70, 1.02)	0.387
DVR, *median (IQR)*	0.23 (0.08, 0.43)	0.37 (0.19, 0.50)	0.16 (0.06, 0.28)	<0.001*
cDVR, *median (IQR)*	0.02 (0.01, 0.06)	0.02 (0.01, 0.05)	0.02 (0.01, 0.07)	0.725
rDV, *median (IQR)*	6.70 (2.00, 17.70)	11.56 (4.14, 28.01)	3.96 (1.51, 11.03)	<0.001*
Venous level collateral				
DVO score, *median (IQR)*	4.00 (3.00, 5.00)	3.00 (2.00, 3.25)	5.00 (4.00, 5.00)	<0.001*
SVO score, *median (IQR)*	4.00 (3.00, 5.00)	3.00 (2.00, 4.00)	5.00 (4.00, 5.00)	<0.001*

mCTA, multi-phase computed tomography angiography; AIS, acute ischemic stroke; CR, collateral ratio; IQR, interquartile range; DVR, delayed vessel ratio; cDVR, contralateral DVR; rDV, relative delayed vessel; DVO, deep cerebral venous outflow; SVO, superficial cerebral venous outflow. #Functional independence is defined as modified Rankin scale score = 0 ~ 2. *Significant differences.

**Table 3:** The association between collateral parameters derived from color-coded mCTA maps and functional independence^#^ of AIS patients.

Parameters	Unadjusted OR (95% CI)	*p*-value	Adjusted^##^ OR (95% CI)	*p*-value
CR	0.90 (0.59 ~ 1.37)	0.619	0.83 (0.53 ~ 1.30)	0.411
DVR	0.01 (0.00 ~ 0.08)	<0.001*	0.04 (0.01 ~ 0.26)	<0.001*
cDVR	46.90 (0.21 ~ 10527.55)	0.164	210.78 (0.47 ~ 95250.08)	0.086
rDV	0.98 (0.97 ~ 0.99)	0.013*	0.99 (0.97 ~ 1.00)	0.094
DVO score	3.53 (2.41 ~ 5.18)	<0.001*	3.06 (2.06 ~ 4.53)	<0.001*
SVO score	2.76 (1.96 ~ 3.90)	<0.001*	2.34 (1.64 ~ 3.34)	<0.001*

mCTA, multi-phase computed tomography angiography; AIS, acute ischemic stroke; OR, odds ratio; 95% CI, 95% confidence interval; CR, collateral ratio; IQR, interquartile range; DVR, delayed vessel ratio; cDVR, contralateral DVR; rDV, relative delayed vessel; DVO, deep cerebral venous outflow; SVO, superficial cerebral venous outflow. #Functional independence was defined as modified Rankin scale score = 0 ~ 2. ##Adjusted for the propensity scores based on baseline National Institutes of Health Stroke Scale score, endovascular thrombectomy, and symptomatic intracranial hemorrhage. *Significant differences.

**Table 4:** The association between collateral parameters derived from color-coded mCTA maps and excellent recovery^#^ of AIS patients.

Parameters	Unadjusted OR (95% CI)	*p*-value	Adjusted^##^ OR (95% CI)	*p*-value
CR	0.65 (0.22 ~ 1.91)	0.435	0.63 (0.16 ~ 2.42)	0.503
DVR	0.00 (0.00 ~ 0.01)	<0.001*	0.00 (0.00 ~ 0.03)	<0.001*
cDVR	0.00 (0.00 ~ 2.16)	0.084	0.00 (0.00 ~ 1.87)	0.069
rDV	0.97 (0.95 ~ 0.99)	0.021*	0.99 (0.97 ~ 1.01)	0.196
DVO score	2.20 (1.62 ~ 2.99)	<0.001*	2.00 (1.40 ~ 2.85)	<0.001*
SVO score	1.91 (1.41 ~ 2.59)	<0.001*	1.68 (1.15 ~ 2.44)	0.007*

mCTA, multi-phase computed tomography angiography; AIS, acute ischemic stroke; OR, odds ratio; 95% CI, 95% confidence interval; CR, collateral ratio; IQR, interquartile range; DVR, delayed vessel ratio; cDVR, contralateral DVR; DVO, deep cerebral venous outflow; SVO, superficial cerebral venous outflow. #Excellent recovery was defined as modified Rankin scale score = 0 ~ 1. ##Adjusted for the propensity scores based on baseline National Institutes of Health Stroke Scale score, hypertension, and endovascular thrombectomy. *Significant differences.

Moreover, the venous level collateral circulation, measured using the DVO score and SVO score, also showed significant differences between the functionally dependent and independent groups (Table 2). Both the median DVO score (3 points versus 5 points) and median SVO score (3 points versus 5 points) were lower in the functionally dependent group compared to the functionally independent group (both *p* < 0.001). Both high DVO score and high SVO score with and without adjustment were predictive factors for functional dependence and excellent recovery (Tables 3, 4).

### 3.3 Prediction of functional independence

The ROC analysis further confirmed the predictive value of arterial level collateral parameters alone (based on DVR) on functional outcomes. Specifically, at a cut-off value of 0.251, the DVR alone exhibited a sensitivity of 0.67, a specificity of 0.71 and an AUC of 0.73. On the other hand, the venous level collateral score (DVO score) displayed more promising results, with an AUC of 0.86 (Table 5).

**Table 5:** Performance of prediction models based on color-coded mCTA maps for functional independence and excellent recovery^#^ of AIS patients.

Prediction models	AUC (95%CI)	Accuracy (95%CI)	Sensitivity (95%CI)	Specificity (95%CI)	PPV (95%CI)	NPV (95%CI)
Prediction for functional independence						
Arterial level collateral (DVR)	0.73 (0.66–0.81)	0.69 (0.62–0.76)	0.67 (0.57–0.78)	0.71 (0.62–0.80)	0.65 (0.55–0.76)	0.73 (0.63–0.82)
Venous level collateral (DVO score)	0.86 (0.80–0.91)	0.80 (0.74–0.86)	0.75 (0.65–0.85)	0.85 (0.78–0.92)	0.80 (0.71–0.90)	0.81 (0.73–0.88)
Adjusted arterial level collateral^##^	0.81 (0.74–0.88)	0.78 (0.70–0.84)	0.78 (0.68–0.87)	0.77 (0.69–0.86)	0.74 (0.64–0.83)	0.81 (0.73–0.89)
Adjusted venous level collateral^##^	0.88 (0.83–0.93)	0.83 (0.76–0.88)	0.87 (0.79–0.94)	0.80 (0.71–0.88)	0.78 (0.69–0.87)	0.88 (0.81–0.95)
Prediction for excellent recovery						
Arterial level collateral (DVR)	0.80 (0.73–0.87)	0.73 (0.65–0.79)	0.65 (0.56–0.74)	0.88 (0.80–0.96)	0.91 (0.85–0.97)	0.57 (0.46–0.67)
Venous level collateral (DVO score)	0.76 (0.69–0.84)	0.67 (0.59–0.74)	0.57 (0.48–0.66)	0.86 (0.77–0.95)	0.89 (0.81–0.96)	0.51 (0.41–0.61)
Adjusted arterial level collateral^###^	0.90 (0.85–0.95)	0.85 (0.78–0.90)	0.82 (0.75–0.89)	0.90 (0.82–0.97)	0.94 (0.89–0.99)	0.72 (0.62–0.83)
Adjusted venous level collateral^###^	0.88 (0.83–0.93)	0.80 (0.73–0.86)	0.79 (0.72–0.87)	0.81 (0.71–0.91)	0.89 (0.83–0.95)	0.67 (0.56–0.78)

AIS, acute ischemic stroke; AUC, area under the receiver operating characteristic curve; CI, confidence interval; PPV, positive predictive value; NPV, negative predictive value; CR, collateral ratio; DVR, delayed vessel ratio, DVO, deep cerebral venous outflow; SVO, superficial cerebral venous outflow. # Functional independence was defined as modified Rankin scale (mRS) score = 0 ~ 2; excellent recovery was defined as mRS score = 0 ~ 1. ##Adjusted for the propensity scores based on baseline National Institutes of Health Stroke Scale (NIHSS) score, endovascular thrombectomy (EVT), and symptomatic intracranial hemorrhage. ###Adjusted for the propensity scores based on baseline NIHSS score, hypertension, and EVT.

After adjustment for patients’ baseline NIHSS score, EVT intervention, and SICH events, the performance of the arterial level collateral model (*p* = 0.010) indicated a marked improvement. However, the venous level collateral model did not achieve significant (*p* = 0.084) improvement after such adjustment.

**Figure 2:**
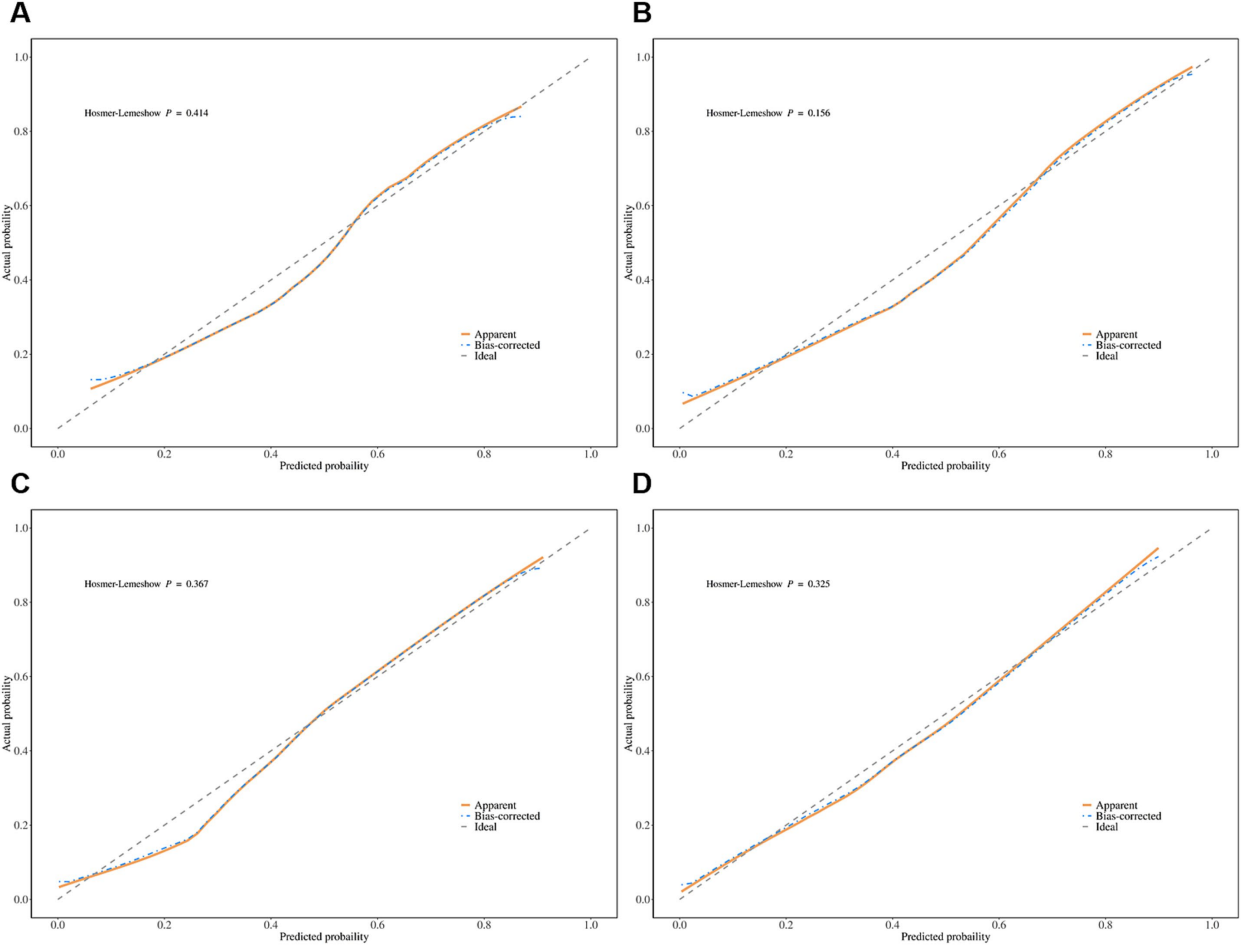
Calibration curve of adjusted arterial level collateral **(A)** and adjusted venous level collateral **(B)** for predicting functional independence. Calibration curve of adjusted arterial level collateral **(C)** and adjusted venous level collateral **(D)** for predicting excellent recovery.

**Figure 3:**
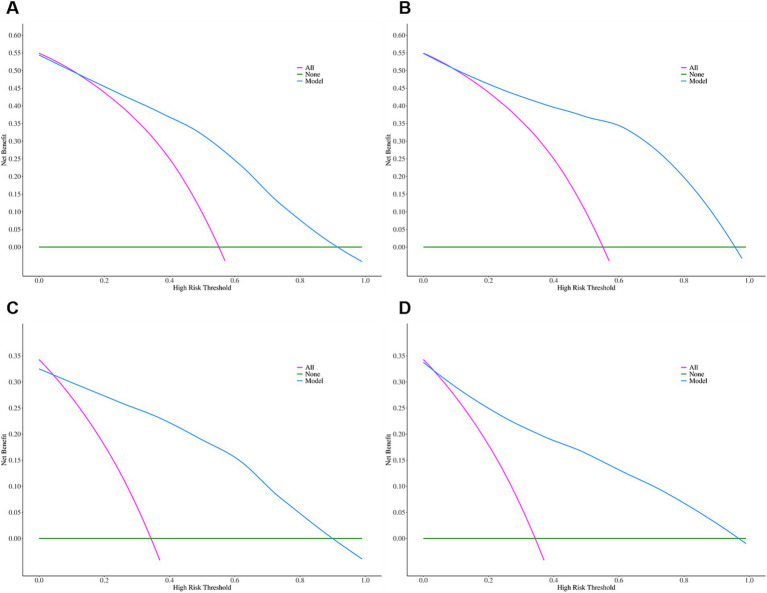
Decision curve analysis of adjusted arterial level collateral **(A)** and adjusted venous level collateral **(B)** for predicting functional independence. Decision curve analysis of adjusted arterial level collateral **(C)** and adjusted venous level collateral **(D)** for predicting excellent recovery.

The calibration curve indicated a satisfactory consistency between the predicted and actual risks for both the unadjusted model and adjusted model in predicting functional independence (Figure 2). The DCA indicated that the model yielded great potential for clinical utility in stroke management and risk prediction (Figure 3).

### 3.4 Prediction of excellent recovery

For the prediction of excellent recovery, the arterial level collateral parameter alone (based on DVR) demonstrated a sensitivity of 0.65 and a specificity of 0.88 at a cut-off value of 0.247, with an AUC of 0.80 in the ROC analysis. On the venous side, the venous level collateral parameter alone (DVO score) showed inferior results, with an AUC of 0.76 (Table 5).

After adjustment for patients’ sex, baseline NIHSS score, comorbidities of hypertension and intervention of EVT, the arterial level collateral model (*p* < 0.001) and venous level collateral model (*p* < 0.001) indicated a significantly better performance.

The calibration curve indicated good precision for each model in predicting excellent recovery, while the DCA showed a good clinical net benefit for stroke management (Figures 2, 3).

## 4 Discussion

Our study indicates that a user-friendly collateral assessment, which integrates both quantitative arterial-level and semi-quantitative venous-level components, shows potential as a visual predictor of functional independence and excellent recovery in AIS patients. Our research offers a quantitative perspective on the conventional ASPECT scoring scheme utilizing grayscale CT images. The substantial diagnostic efficacy observed for the quantitative arterial collateral parameters, particularly the DVR, underscores their potential utility in clinical practice.

Our research has established a significant correlation between the admission NIHSS score and independent neurologic functional outcomes, providing valuable insights for clinical decision-making in high-risk AIS patients. Likewise, Mihindu et al. demonstrated that individuals with moderate to severe strokes (NIHSS score >10) did not achieve favorable clinical outcomes following urgent interventions (24, 25). Our study also suggests that the proportion of males is higher in both groups. This finding is consistent with the results of Voigt et al., who examined sex differences in intracranial and extracranial atherosclerosis in AIS patients and found that males accounted for a relatively high proportion of the total cohort (26). Additionally, our study revealed that hypertension is the most common condition in AIS patients with varying severity. Thus, blood pressure management is critical for primary prevention of AIS and hemorrhagic stroke as recommended by recent guideline (27).

In contrast to prior subjective visual assessments of cmCTA maps (21, 22), our study utilized comprehensive quantitative or semi-quantitative analysis to evaluate collateral circulation at both arterial and venous levels. This approach provided significant insights into the status of the collateral network. Our findings showed that patients with strong arterial level collateral, as indicated by DVR and rDV parameters, had more favorable clinical outcomes compared to those with limited or absent collateral circulation. This result aligns with prior research utilizing varied qualitative or semi-quantitative methodologies, underscoring the importance of arterial level collateral in preserving cerebral perfusion following AIS (12, 20, 28). Furthermore, our results suggest that venous outflow, as assessed through DVO and SVO scores, significantly influences the microvascular perfusion and functional outcomes of AIS patients, which is in line with prior research (10, 11, 14). Additionally, we identified several factors, such as baseline stroke severity, clinical comorbidities, therapeutic methodology and SICH events, that influence the development and maintenance of arterial or venous level collateral, based on different endpoint indicators (functional independence or excellent recovery). Similar to the prediction model construction protocol of our study, stroke researchers advocate for adjusting models to account for baseline stroke severity, such as the NIHSS score, in clinical predictions (24).

In our study, we preliminarily developed a series of prediction models for functional independence post-AIS. These models incorporated both collateral parameters (DVR and DVO score) and clinical factors to achieve a high level of precision in forecasting functional outcomes, resulting in an AUC range of 0.73 to 0.88. The most influential predictor of functional independence was found to be the adjusted venous level collateral. The negative predictive value associated with this predictor was as high as 0.88, indicating that the adjusted venous-collateral-based model provided valuable insights for excluding functional independence and identifying potential risk cohorts for AIS patients management.

Moreover, we conducted a detailed examination of the ability to predict excellent recovery following AIS, characterized by minimal or absent residual disability. Our predictive model, developed using a framework akin to the functional independence model, demonstrated encouraging outcomes. The AUC for forecasting excellent recovery utilizing collateral parameters (DVR or DVO score) ranged from 0.76 to 0.80, signifying a robust capacity for accurate prediction. Notably, after adjusting for propensity scores, arterial collateral circulation emerged as the most influential predictor. Our study revealed that the positive predictive value of adjusted arterial collateral level was 0.90 in predicting excellent recovery, suggesting a high likelihood of accurately identifying patients who would achieve this favorable outcome after receiving timely treatment.

Similar to our research, Su et al. focused on deriving an optimal CR measurement on perfusion CT and mCTA for AIS patients. However, the CR assessment in the previous study is dependent on the timing of acquisition or the specific phase selection (17). Boers introduced a comparable automated quantitative collateral scoring method in AIS patients, which provided as a reliable and user-independent measure of the collateral capacity on baseline CTA and has the potential to augment the triage of EVT therapy. Even so, the relation of sCTA-based quantitative collateral score and digital subtraction angiography-based collateral scores was expected to be weak, indicating sCTA was limited in its ability to quantitatively evaluate the dynamic cerebral circulation (18).

Unexpectedly, the CR ratio did not demonstrate a statistically significant association with the prediction of functional independence and excellent recovery. In contrast to prior research (17, 18), the CR in our study was derived from an extensive analysis of the fusion images from multiple phases rather than single-phase images, encompassing vessels that exhibited marked enhancement in the peak phase as well as those with delayed enhancement. This methodology may result in an overestimation of the CR level on the affected side due to the inclusion of delayed enhanced vessels on that side, which diminishes the bilateral differences of enhanced vessels. Consequently, a focused analysis of delayed vessels (such as DVR) may offer a more significant approach for quantitatively assessing collateral circulation on cmCTA maps within the ACA and ASPECT regions.

The findings presented above offer clinicians a valuable tool, specifically an adjusted venous collateral model, for predicting functional independence in stroke patients. This tool could be operationalized into routine stroke workflows and aid in identifying patients who are unlikely to have a favorable clinical outcome, allowing for early intervention, individualized nursing, close monitoring, and timely rehabilitation planning. The use of the venous collateral score on cmCTA, as demonstrated in this study, may decrease the need for EVT interventions due to inadequate patient selection, as seen in a previous study (29). On the other hand, a specific model, such as an adjusted arterial collateral model, can assist in clinical decision-making by identifying patients who are most likely to benefit from aggressive therapies, such as EVT, aimed at improving collateral circulation. Our results support the use of cmCTA as an initial assessment for all AIS patients (8), as it may increase the number of suitable candidates for EVT and potentially lead to improved outcomes. As a whole, our findings highlight the importance of early assessment and optimization of both arterial and venous collateral levels on cmCTA in promoting ideal recovery after stroke.

This study has several limitations that should be taken into consideration. Firstly, the data-set was collected from a single center, which may limit the generalizability of the results. To improve the reliability of the findings, it is recommended to conduct multi-center validation. Secondly, the semi-automated image reconstruction and collateral scoring method employed in this study may introduce regional variations or heterogeneity. Further technical refinements are necessary, especially in the automation of standardized reconstruction and image segmentation procedures. These enhancements are critical for improving intra-rater consistency and reliability in interpreting cmCTA maps. Ultimately, the venous collateral scoring method utilized in this study remains subjective and may be influenced by variations in the cerebral venous system or inherent artifacts of cmCTA map. Therefore, it is crucial to validate or improve this method in larger clinical studies.

## 5 Conclusion

In summary, our findings suggest that both quantitative arterial collateral parameters and semi-quantitative venous collateral score have significant predictive value for functional independence and excellent recovery in AIS patients. Additionally, our results indicate that quantitative analysis of cmCTA, with a focus on DVR, has the potential to realize the precise quantification of ASPECT score, improve clinical decision-making, and optimize the management of AIS patients in the future.

## Data Availability

The raw data supporting the conclusions of this article will be made available by the authors, without undue reservation.
