# Supplementary material for: Quantitative insights into stroke recovery utilizing delayed vessel ratio from color-coded multiphase computed tomography angiography
Source: Front Neurol. 2025 Jun 18;16:1568717. doi: 10.3389/fneur.2025.1568717 (PMC12214355; doi:10.3389/fneur.2025.1568717)
Supplement: Supplementary file 1 [file Table_1.docx]

**Semi-quantitative venous level collateral score**

Using a 6-point scale ranging from 0 (worst) to 6 (best), two radiologists independently assessed collateral circulation at the venous level on the ColorViz map without knowing the patient's prognosis. In cases of inconsistent results, discrepancies were resolved through negotiation. Specifically, the degree of venous filling (ranging from 0 to 3 points) and the degree of phase delay (ranging from 0 to 3 points) were evaluated separately, and the combined total score was calculated. Scores for deep venous outflow (DVO) and superficial venous outflow (SVO) were assessed respectively.

The specific scoring method for evaluating the degree of venous filling and the degree of phase delay was as follows:

1. **Degree of venous filling (venous filling score)**

(1) 3 points: Venous filling was robust and symmetrical compared to the contralateral side, with no obvious filling defects, irrespective of the degree of blood-flow filling delay.

(2) 2 points: Venous filling was moderate compared to the contralateral side, with focal filling defects present, irrespective of the degree of blood-flow filling delay.

(3) 1 point: Venous filling was poor compared to the contralateral side, with multiple or diffuse filling defects, irrespective of the degree of blood-flow filling delay.

(4) 0 points: Virtually no venous filling was observed compared to the contralateral side.

**2. Degree of phase delay (venous blood-flow score)**

(1) 3 points: There was no significant delay in venous blood flow (indicated by red vessels on the ColorViz fusion maps).

(2) 2 points: At least 50% of the venous blood flow showed no significant delay (red vessels on the ColorViz fusion maps), but some areas exhibited delayed venous blood flow (green or blue vessels on the ColorViz fusion maps).

(3) 1 point: More than 50% of the venous blood flow was delayed by one phase (green vessels on the ColorViz fusion maps).

(4) 0 points: At least 50% of the venous blood flow was delayed by two phases (blue vessels on the ColorViz fusion maps), or there was virtually no venous filling.
